# *Coxiella burnetii* effector CelF reprograms host proteostasis via competitive disruption of the UBQLN1-PSMD4 axis to stabilize autophagic SNARE SNAP29 and modulate lysosomal metabolic maturation

**DOI:** 10.3389/fphys.2026.1957980

**Published:** 2026-10-14

**Authors:** Shan Zhang, Yufei Jiang, Yinhui Lin, Xiaoxiao Chen, Yuchen Zhang, Yonghui Yu, Xuan OuYang, Dongsheng Zhou, Yajun Song, Jun Jiao

**Affiliations:** State Key Laboratory of Pathogen and Biosecurity, Academy of Military Medical Sciences, Beijing, China

**Keywords:** autophagic flux, intracellular infection metabolism, lysosomal metabolism, protein homeostasis, SNARE proteostasis, ubiquitin-proteasome system, UBQLN1

## Abstract

**Introduction:**

The ubiquitin-proteasome system (UPS) and autophagy-lysosome pathway constitute the core proteolytic metabolic networks that maintain cellular protein homeostasis (proteostasis) and organelle metabolic identity. Intracellular pathogens frequently reprogram host metabolic pathways to establish replicative niches, yet how they manipulate proteostatic metabolism to remodel organelle maturation remains incompletely understood.

**Methods:**

CelF secretion was confirmed by TEM translocation assay. CRISPR-dCas9 was used to generate CelF knockdown and complementation strains, with celF transcription verified by RT-qPCR. Cell infection and SCID mouse models were used to assess intracellular survival, Coxiella-containing vacuole (CCV) maturation, and virulence of Coxiella burnetii. The effect of CelF on late autophagy was examined by dual-fluorescence LC3 and Western blotting. Then, UBQLN1 was identified as a CelF-interacting host protein by co-IP and pull-down, followed by truncation mapping. Finally, co-IP, UBQLN1 knockdown, Western blotting and MST assays were used to analyze the effect of CelF on SNARE assembly, SNAP29 degradation and ubiquitination, and competitive binding of CelF and PSMD4 to UBQLN1.

**Results:**

CelF depletion severely impaired the metabolic maturation of CCV, suppressed bacterial proliferation in macrophages, and attenuated virulence in vivo. Mechanistically, CelF acted as a molecular decoy that directly bound the ubiquitin-like (UBL) domain of the proteasomal shuttle factor UBQLN1, competitively displacing the proteasome subunit PSMD4. This competitive interference blocked UBQLN1-mediated proteasomal delivery of SNAP29 without altering its ubiquitination status, thereby enhancing SNAP29 protein stability and promoting assembly of the STX17-SNAP29-VAMP8 fusogenic complex. The augmented SNARE activity drove autophagosome-lysosome fusion, sustained autophagic flux, and facilitated lysosomal metabolic remodeling to support CCV biogenesis.

**Discussion:**

Unlike canonical pathogen-encoded UPS modulators that function as E3 ligases or deubiquitinases, CelF operates purely through competitive protein-protein interaction, representing a previously unrecognized paradigm for microbial manipulation of host proteostatic metabolism. Our findings reveal that intracellular pathogens can actively orchestrate organelle metabolic identity by rewiring host proteostasis, and identify the UBQLN1-PSMD4-SNAP29 axis as a novel regulatory checkpoint for SNARE proteostasis and lysosomal metabolic maturation.

## Introduction

Cellular metabolic homeostasis relies on tightly regulated protein turnover and organelle communication. The ubiquitin-proteasome system (UPS) and the autophagy-lysosome pathway represent two major proteolytic metabolic axes that govern protein quality control, organelle renewal, and energy recycling in eukaryotic cells (Pohl and Dikic, 2019). Disruption of these metabolic systems underlies a broad spectrum of human diseases, from neurodegeneration to metabolic disorders (Menzies et al., 2017). At the cellular level, lysosomes serve as central metabolic organelles responsible for the degradation and recycling of macromolecules, and their functional maturation is tightly coupled to membrane fusion events mediated by soluble N-ethylmaleimide-sensitive factor attachment protein receptor (SNARE) complexes (Chen and Scheller, 2001; Tian et al., 2021). As core machinery for intracellular membrane fusion, SNARE proteins not only mediate organelle metabolic communication but also determine the metabolic identity and degradative capacity of lysosomes. However, the metabolic regulatory mechanisms that control SNARE protein stability and turnover remain incompletely characterized.

Intracellular bacterial pathogens have evolved sophisticated strategies to reprogram host cellular metabolism and remodel organelle environments for survival (Asrat et al., 2014). Unlike most intravacuolar pathogens that block phagolysosomal maturation to avoid lysosomal degradation, *Coxiella burnetii*, the causative agent of Q fever, actively promotes fusion of its nascent phagosome with lysosomes to establish a replication-permissive *Coxiella*-containing vacuole (CCV) (Voth and Heinzen, 2007; Wang et al., 2024). The CCV is a modified phagolysosome with characteristic lysosomal metabolic features, including acidic pH, hydrolytic enzymes, and lysosome-associated membrane proteins (Voth and Heinzen, 2007; Newton et al., 2020; Bird et al., 2025). Accumulating evidence indicates that CCV biogenesis is metabolically intertwined with the host autophagic pathway, and *C. burnetii* hijacks autophagic membranes to support CCV expansion via autophagosome-lysosome fusion (Gutierrez et al., 2005; Kohler and Roy, 2015; Thomas et al., 2020). This unique reliance on lysosomal and autophagic metabolic pathways raises a fundamental question in both microbial pathogenesis and cellular metabolic physiology: how does an intracellular bacterium actively modulate host proteolytic metabolism to drive organelle maturation without disrupting the metabolic homeostasis required for its own replication?

The Dot/Icm type IV secretion system (T4SS) is essential for *C. burnetii* to manipulate host vesicular trafficking and CCV fusogenicity (Carey et al., 2011; Newton and Roy, 2011). Over 140 candidate T4SS effectors have been identified, which collectively modulate host signaling, cytoskeletal dynamics, and membrane metabolism (Larson et al., 2016). Despite their critical roles, the molecular mechanisms by which these effectors interface with host proteostatic metabolism to control SNARE-dependent membrane fusion remain largely elusive. The autophagosome-lysosome fusion event is executed by a ternary SNARE complex composed of autophagosome-resident STX17, cytosolic adaptor SNAP29, and lysosome-resident VAMP8 (Itakura et al., 2012; Jian et al., 2024). While the function of this complex in autophagy is well established, its metabolic regulation at the protein stability level is poorly defined. Given that the UPS controls the turnover of most cellular proteins (Rock et al., 1994), we hypothesized that *C. burnetii* may target UPS-mediated SNARE proteostasis to tune lysosomal metabolic maturation.

Ubiquilin-1 (UBQLN1) is a proteasomal shuttle factor that bridges ubiquitinated substrates to the 26S proteasome via its N-terminal ubiquitin-like (UBL) domain, which interacts with the proteasome subunit PSMD4, and its C-terminal ubiquitin-associated (UBA) domain, which binds polyubiquitinated cargo (Ko et al., 2004; Kurlawala et al., 2017). As a key component of the proteostatic metabolic network, UBQLN1 maintains cellular protein quality control and has been implicated in metabolic and neurodegenerative diseases (Takalo et al., 2013; Zheng et al., 2020). However, its role in regulating SNARE protein turnover and its involvement in pathogen-induced metabolic reprogramming remain unexplored.

Our previous protein-protein interaction network analysis suggested that the *C. burnetii* protein CBU1825 (designated CelF, *Coxiella* effector for autophagosome-lysosome fusion) is functionally associated with autophagy and proteasomal pathways. In this study, we demonstrate that CelF is a bona fide T4SS effector that functions as a non-enzymatic molecular decoy to disrupt the UBQLN1-PSMD4 interaction. By competitively occupying the UBL domain of UBQLN1, CelF selectively inhibits proteasomal degradation of SNAP29, enhances SNARE complex assembly, and promotes autophagosome-lysosome fusion, thereby driving lysosomal metabolic maturation of the CCV. Our findings not only reveal a novel microbial strategy for host metabolic subversion but also identify the UBQLN1-PSMD4 axis as a physiological regulatory checkpoint for SNARE proteostasis and lysosomal metabolic function.

## Results

### CelF is a T4SS effector required for lysosomal metabolic maturation of the CCV and full bacterial virulence

We generated NMII and dotA::Tn strains expressing β-lactamase-fused CelF (**Supplementary Figure S1A**). Using a β-lactamase-based assay, we confirmed T4SS-dependent translocation of CelF in C. burnetii (**Figure 1A**), consistent with previous studies (Chen et al., 2010; Carey et al., 2011; Larson et al., 2019).

**Figure 1:**
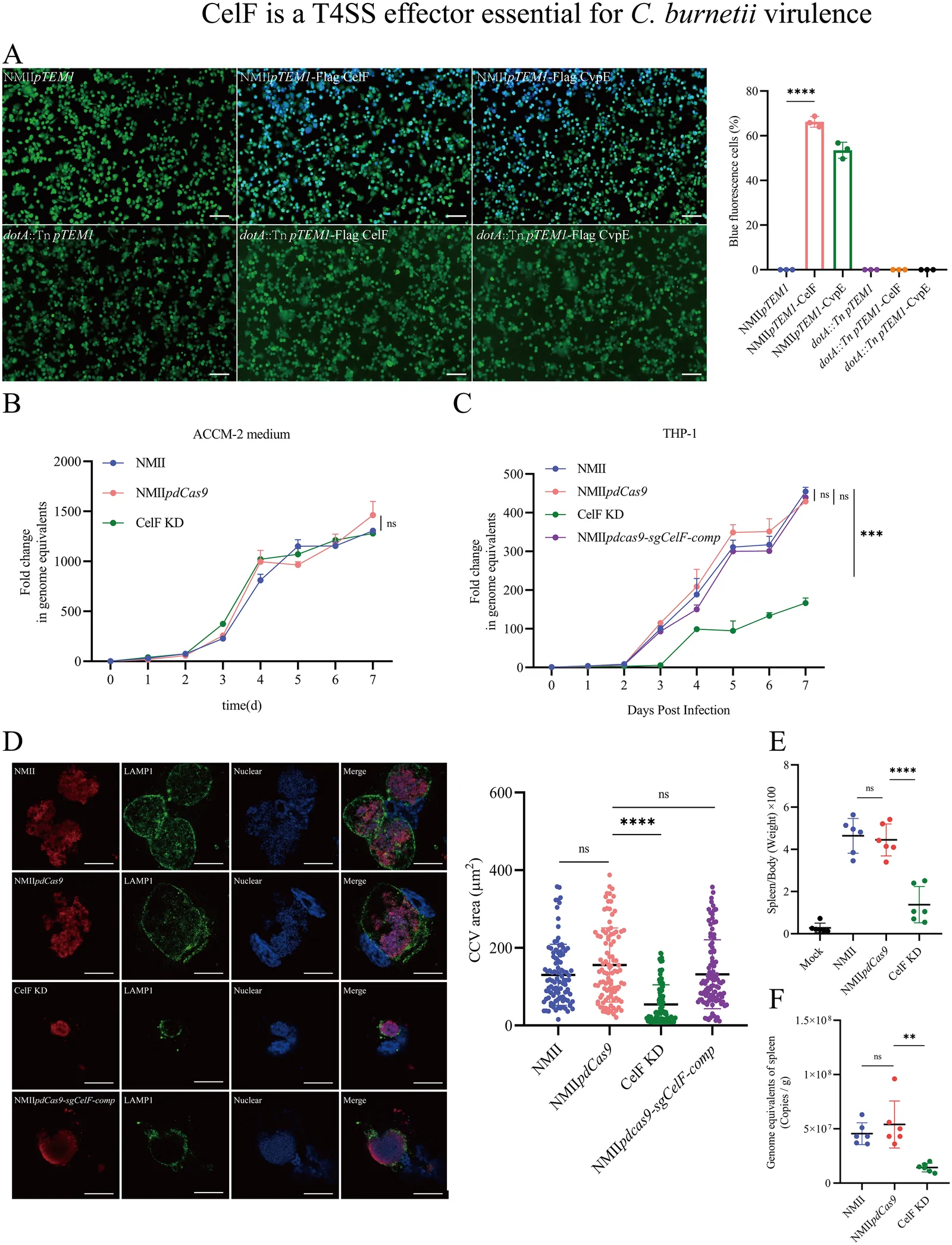
CelF is a T4SS effector required for lysosomal metabolic maturation of the CCV and full bacterial virulence. **(A)** T4SS-dependent translocation of CelF was assessed using a β-lactamase (TEM) reporter assay. Differentiated THP-1 cells were infected with the indicated strains (MOI = 20). Representative fluorescence images and quantification of cells with cleaved CCF4-AM substrate (blue fluorescence) are shown. Scale bar, 100 µm. **(B)** Growth of NMII, NMII*pdCas9*, CelF KD, and NMII*pdCas9*-*sgCelF*-*comp* strains in ACCM-2 axenic medium. Bacterial loads were quantified by qPCR at the indicated time points. **(C)** Intracellular replication of the indicated strains in differentiated THP-1 cells (MOI = 5). Bacterial GEs were quantified by qPCR over 7 days. **(D)** CCV morphology in THP-1 cells infected with the indicated strains (MOI = 20) for 3 days. CCV cross-sectional areas were quantified from representative confocal microscopy images showing bacteria (red), LAMP1 (green), and nuclei (blue). Scale bar, 10 µm. n = 100. **(E, F)** Virulence was assessed in SCID mice infected with the indicated strains. **(E)** Spleen-to-body weight ratios at 14 days post-infection. **(F)** Bacterial loads in spleens. n = 6 mice per group. Significance is indicated as follows: ns, P ≥ 0.05, **P < 0.01, ***P < 0.001, ****P < 0.0001.

We constructed CelF knockdown (KD) strain (NMIIpdCas9-sgCelF) using a modified CRISPRi system, as previously described (Fu et al., 2022; Steiner and Roy, 2024), and a complemented strain (NMIIpdCas9-sgCelF-comp) as described (Zhang et al., 2022). RT-qPCR confirmed that the sgRNA2 knockdown strain, which exhibited the highest knockdown efficiency among three independent sgRNA strains, achieved approximately 90% reduction in transcriptional levels of celF compared with the wild-type and control strains. This reduction was restored in the complemented strain (**Supplementary Figure S1B**). The sgRNA2 knockdown strain was therefore selected for all subsequent experiments. To assess the effect of CelF on bacterial replication, we infected THP-1 cells with C. burnetii and quantified bacterial loads via quantitative PCR (qPCR). The CelF KD strain grew normally in ACCM-2 medium (**Figure 1B**) but was significantly impaired in THP-1 cells (**Figure 1C**). Complementation with wild-type CelF restored intracellular replication (**Figure 1C**). The same results were obtained in HeLa and BGMK cells (**Supplementary Figures S1C, D**). We next examined the vacuole morphology. NMII and NMIIpdCas9 control strains formed large LAMP1-positive vacuoles, whereas CelF KD produced only small vacuoles; complementation partially restored CCV size (**Figure 1D**).

To assess the role of CelF in the virulence of C. burnetii, SCID mice (van Schaik et al., 2017) were infected with the NMII, NMIIpdCas9, or CelF KD strains. Spleens were harvested at 2 weeks for weight and bacterial load analysis. Infection with the NMII strain resulted in significant splenomegaly compared with uninfected controls (**Supplementary Figure S1E**). Notably, the spleen-to-body weight ratios of mice infected with the CelF KD strain were significantly lower than those of mice infected with either the NMII or NMIIpdCas9 strains (**Figure 1E**). Correspondingly, bacterial loads in the spleens of CelF KD strain-infected mice were markedly reduced compared to those in mice infected with the other two strains (**Figure 1F**). Together, these findings demonstrate that CelF is an essential virulence factor required for full C. burnetii virulence in the SCID mouse model.

### CelF enhances autophagic flux and counteracts lps-induced metabolic blockade of autophagosome-lysosome fusion

Previous studies have shown that LPS can block the late stage of autophagy (Barry et al., 2012; Li et al., 2023). To confirm this, we utilized the RFP-GFP-LC3 assay to track the autophagic flux. In this system, the acidic environment of the autolysosome quenches the GFP signal, so red-only puncta represent autolysosomes, while yellow puncta (GFP^+^RFP^+^) represent autophagosomes (Kimura et al., 2007). Both E. coli and C. burnetii LPS induced accumulation of yellow LC3 puncta compared with controls, indicating blocked autophagosome-lysosome fusion (**Supplementary Figure S2A**). The colocalization of endogenous LC3 with the lysosomal marker LAMP1 was significantly decreased after E. coli or C. burnetii LPS treatment in HeLa cells (**Supplementary Figure S2B**). Collectively, these data demonstrate that both LPS sources block autophagosome-lysosome fusion in host cells. Based on this functional equivalence, and to enable large-scale biochemical characterization, we used commercially available E. coli LPS as a surrogate for the following mechanistic experiments.

To assess the relationship between autophagy and C. burnetii replication, we treated cells with rapamycin (enhanced proliferation) or bafilomycin A1 (suppressed replication in a dose-dependent manner) (**Figure 2A**). Given our previous PPI network analysis linking CelF to autophagy (Fu et al., 2022), we next investigated whether CelF influences autophagic flux. CelF-expressing cells showed decreased SQSTM1/p62 and LC3-II levels compared with controls (**Figures 2B–D**). To distinguish between reduced autophagosome formation and enhanced lysosomal degradation, we treated cells with the lysosomal inhibitor bafilomycin A1 (BafA1). In control cells, BafA1 treatment caused accumulation of SQSTM1/p62 and LC3-II, as expected when lysosomal degradation is blocked. In CelF-expressing cells, however, even in the presence of BafA1, SQSTM1/p62 and LC3-II levels, as well as the LC3-II/LC3-I ratio, remained significantly lower than in BafA1-treated control cells (**Figures 2B–D**). This indicates that CelF enhances autophagic flux upstream of lysosomal degradation.

**Figure 2:**
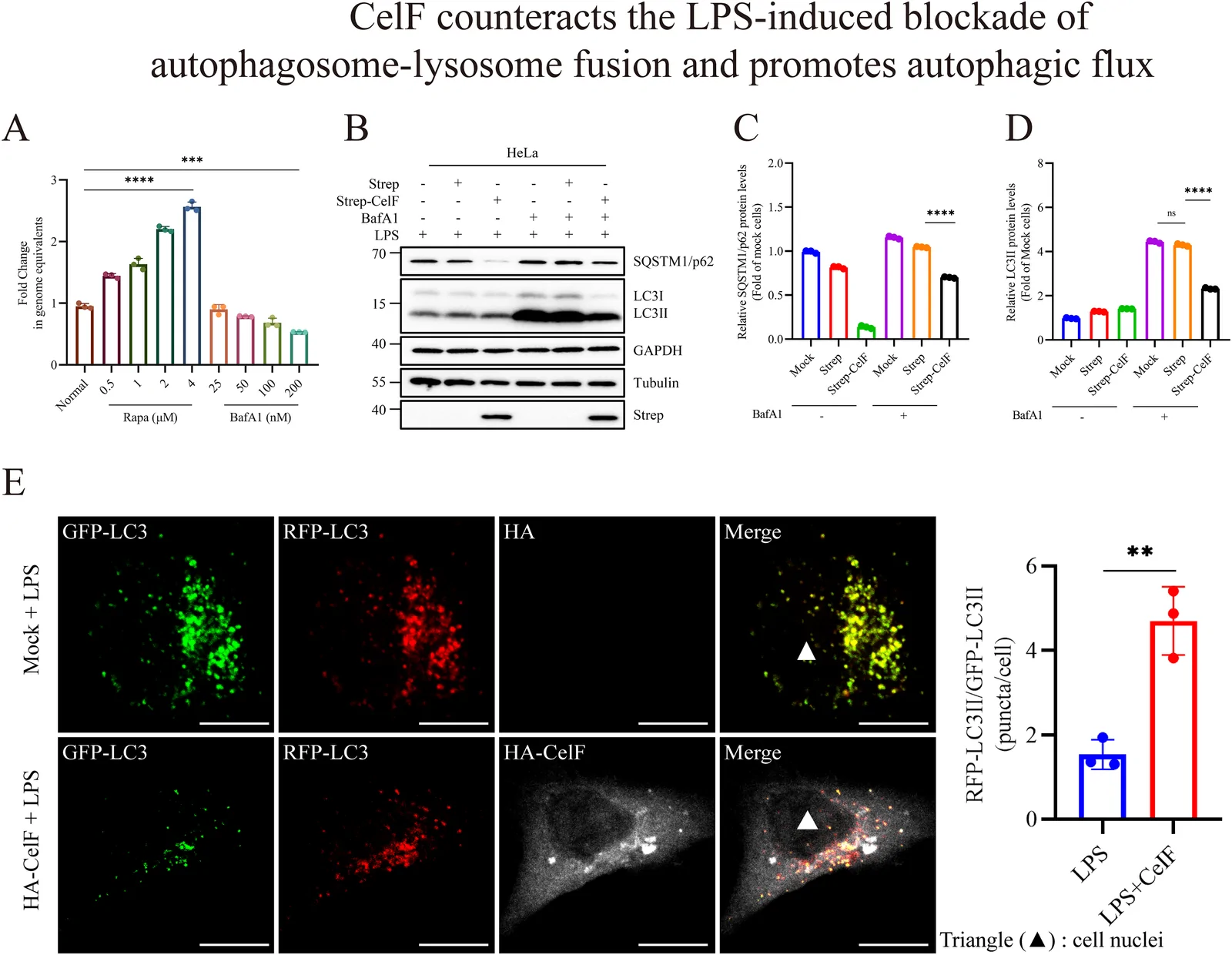
CelF enhances autophagic flux and counteracts LPS-induced metabolic blockade of autophagosome-lysosome fusion. **(A)** Intracellular replication of *C. burnetii* in differentiated THP-1 cells treated with the indicated concentrations of rapamycin or bafilomycin A1 (BafA1). Bacterial GEs were quantified at 4 days post-infection. **(B–D)** Autophagic flux analysis in HeLa cells treated with *E. coli* LPS, transfected with Strep-tagged CelF or an empty vector, and treated with BafA1 (50 nM) or DMSO for 4 h. **(B)** Representative immunoblots of LC3 and SQSTM1/p62. **(C, D)** Densitometric quantification of SQSTM1/p62 levels **(C)** and the LC3-II/LC3-I ratio **(D)**. GAPDH served as a loading control. **(E)** HeLa cells co-transfected with RFP-GFP-LC3 and HA-tagged CelF were treated with *E. coli* LPS. Representative confocal images showing autophagosomes (yellow puncta, GFP^+^RFP^+^) and autolysosomes (red-only puncta, RFP^+^). The number of red-only LC3 puncta and yellow LC3 puncta per cell were quantified. Scale bar, 10 µm. Data are representative of three independent experiments, and bars represent the mean ± SD from three independent experiments. Significance is indicated as follows: ns, *P* ≥ 0.05, **P < 0.01, ***P < 0.001, ****P < 0.0001.

We then investigated whether CelF counteracts the LPS-induced blockade of autophagosome-lysosome fusion. Upon LPS treatment, CelF expression increased the number of red-only LC3 puncta (**Figure 2E**). Quantification of both populations showed that CelF increased autolysosomes (GFP^-^RFP^+^) without reducing total autophagosomes (GFP^+^RFP^+^), indicating that CelF facilitates autophagosome-lysosome fusion and counteracts LPS blockade.

### CelF directly interacts with the UBL domain of the proteasomal shuttle factor UBQLN1

Our previous AP-MS screen identified 1062 CelF-interacting host proteins, with significant enrichment in UPS, autophagy-lysosome, and membrane trafficking pathways (Fu et al., 2022). We applied the MiST algorithm to prioritize high-confidence hits (Verschueren et al., 2015; Penn et al., 2018), yielding 12 candidates (SARS, PHB, Rab11a, TFRC, DNM1L, CYB5R3, COPS5, VDAC3, CD81, ATP5F1, SPAG9, and UBQLN1). UBQLN1 was selected for further analysis, as its biological function aligns perfectly with the cellular processes regulated by CelF, including autophagy and SNARE regulation (Li et al., 2023). UBQLN1 contains a ubiquitin-associated (UBA) domain that binds poly-ubiquitin chains on substrates and a ubiquitin-like (UBL) domain that shuttles them to the proteasome for degradation (Kurlawala et al., 2017).

Co-IP confirmed CelF-UBQLN1 interaction in HEK-293T cells (**Figure 3A**), and an in vitro pull-down assay confirmed direct binding (**Figure 3B**). Supplemented parallel co-IP showed that no specific binding signal was detected between CelF and UBQLN4 or PSMD3 (**Supplementary Figure S3A**), indicating that CelF specifically co-precipitated with UBQLN1. Confocal microscopy showed CelF co-localization with UBQLN1 (**Figure 3C**), but not with the endoplasmic reticulum marker Calnexin (**Supplementary Figure S3B**). Unfortunately, we were unable to detect the CelF–UBQLN1 interaction by co-IP under infection conditions (data not shown). This result likely reflects technical limitations including transient or weak binding affinity, altered CelF expression/modification during infection, and insufficient complex stability under standard co-IP conditions.

**Figure 3:**
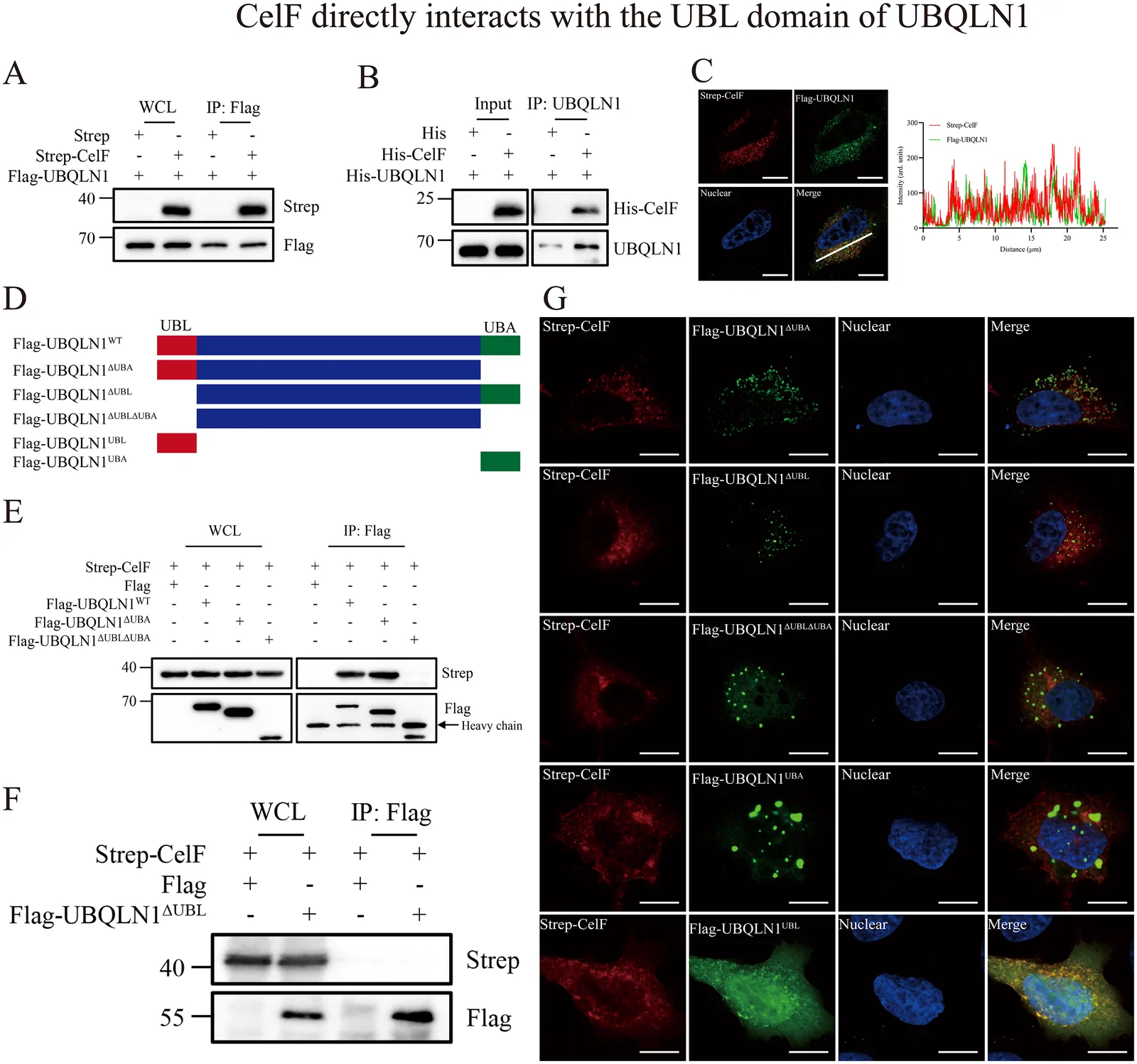
CelF directly interacts with the UBL domain of the proteasomal shuttle factor UBQLN1. **(A)** Co-immunoprecipitation (Co-IP) in HEK-293T cells co-expressing Strep-tagged CelF and Flag-tagged UBQLN1. **(B)** *In vitro* pull-down assay using purified His-tagged UBQLN1 and His-CelF proteins. **(C)** Confocal microscopy and the Pearson’s Correlation Coefficient of HeLa cells expressing mCherry-tagged CelF and Flag-tagged UBQLN1. Scale bars, 10 µm. **(D)** Schematic of UBQLN1 domain architecture and truncation mutants. **(E, F)** Co-IP in HEK-293T cells co-expressing Strep-tagged CelF and Flag-tagged UBQLN1 truncation mutants. **(G)** Confocal microscopy of HeLa cells co-expressing mCherry-tagged CelF and Flag-tagged UBQLN1 truncation mutants. Scale bars, 10 µm.

To map the specific region of UBQLN1 required for CelF binding, we constructed a series of truncation mutants based on its domain architecture (**Figure 3D**). Subsequent co-IP and laser confocal microscopy assays demonstrated that CelF specifically binds to the UBL domain of UBQLN1 (**Figures 3E–G**).

To explore the functional role of UBQLN1 in autophagy, we performed an RFP-GFP-LC3 assay in HeLa cells with stable UBQLN1 knockdown. These cells exhibited a marked accumulation of red-only LC3 puncta compared with control cells (**Supplementary Figure S3C**), indicating enhanced autolysosomal fusion.

Furthermore, UBQLN1 knockdown promoted C. burnetii replication and partially rescued the CelF KD defect (**Supplementary Figure S3D**), suggesting that UBQLN1 is involved in the intracellular survival of the bacterium.

### CelF promotes autophagic SNARE complex assembly in a UBQLN1-dependent manner

We next investigated whether CelF promotes the assembly of the fusogenic SNARE complex. Co-IP experiments revealed that CelF enhanced the interaction between STX17 and SNAP29 upon LPS stimulation, indicating increased formation of the STX17-SNAP29-VAMP8 complex (**Figures 4A–C**). To validate the role of CelF in SNARE complex assembly during infection, we infected HEK-293T cells with NMII, NMIIpdCas9, or CelF KD strains. We found that depletion of CelF in C. burnetii impaired SNARE complex formation (**Figure 4D**).

**Figure 4:**
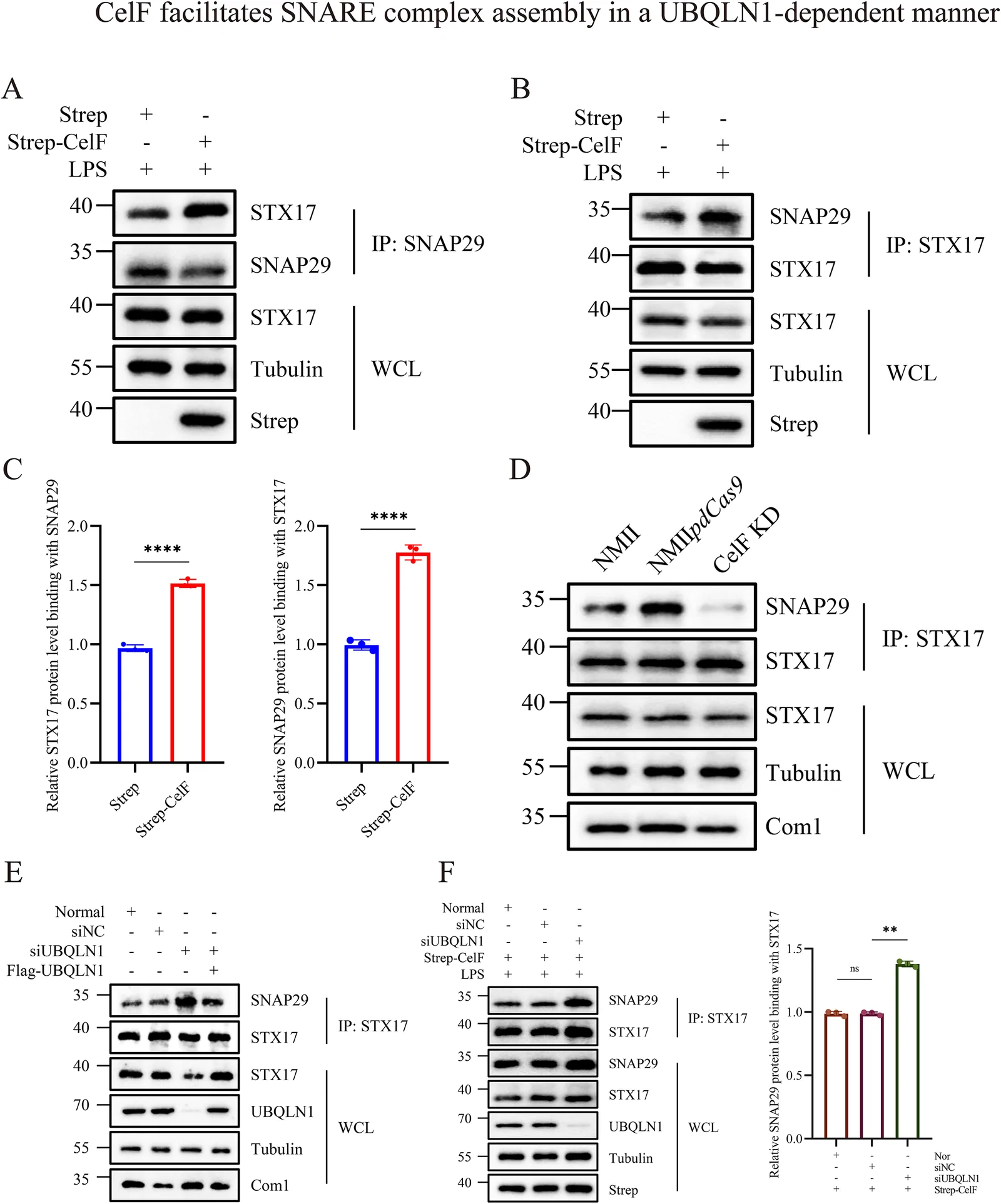
CelF promotes autophagic SNARE complex assembly in a UBQLN1-dependent manner. **(A–C)** HEK-293T cells transfected with Strep-tagged CelF or an empty vector were treated with *E. coli* LPS. Cell lysates were immunoprecipitated with anti-STX17 **(A)** or anti-SNAP29 **(B)** antibodies and immunoblotted for the indicated proteins. **(C)** Densitometric quantification of assembly of SNARE complex. **(D)** HEK-293T cells were infected with the NMII, NMII*pdCas9*, or CelF KD strain (MOI = 20). SNARE complex formation was assessed by Co-IP with an anti-STX17 antibody at 3 days post-infection. **(E)** HEK-293T cells were transfected with control or UBQLN1-targeting siRNA, followed by rescue with Flag-tagged UBQLN1 and infection with the NMII strain. SNARE complex formation was analyzed by Co-IP. **(F)** *E. coli* LPS-treated HEK-293T cells were transfected with control or UBQLN1-targeting siRNA, followed by transfection with Strep-tagged CelF. SNARE complex formation was analyzed by Co-IP. Data are representative of three independent experiments, and bars represent the mean ± SD from three independent experiments. Significance is indicated as follows: ns, *P* ≥ 0.05, **P < 0.01, ****P < 0.0001.

We then asked whether UBQLN1 mediates the regulatory effect of CelF on SNARE complex assembly. Knockdown of UBQLN1 using a short interfering RNA (siRNA) in C. burnetii-infected HEK-293T cells increased SNARE complex assembly (**Figure 4E**). Furthermore, reconstitution of UBQLN1 expression in knockdown cells restored SNARE complex levels to those observed in wild-type conditions (**Figure 4E**). To determine whether CelF alone is sufficient to reproduce this effect through UBQLN1 in the absence of infection, we performed a parallel experiment in CelF-overexpressing HEK-293T cells. Meanwhile, knockdown of UBQLN1 using a short interfering RNA (siRNA) in CelF-expressing HEK-293T cells also increased SNARE complex assembly (**Figure 4F**). Taken together, these results demonstrate that CelF promotes SNARE complex assembly via UBQLN1 during infection.

### CelF stabilizes SNAP29 by antagonizing UPS-mediated proteolytic degradation

Proteins are degraded through two primary pathways: the proteasome pathway or the lysosome pathway (Rusilowicz-Jones et al., 2022). Given that UBQLN1 mediates proteasomal degradation, we investigated whether CelF influences the degradation of the SNARE protein SNAP29.

To determine the degradation pathway for SNAP29, we treated LPS-stimulated HeLa cells with the proteasome inhibitor MG132 or the lysosome inhibitor NH4Cl. Our results showed that SNAP29 reduction was rescued by MG132, but not by NH4Cl, indicating proteasome-dependent degradation of SNAP29 (**Figure 5A**; **Supplementary Figure S4A**).

**Figure 5:**
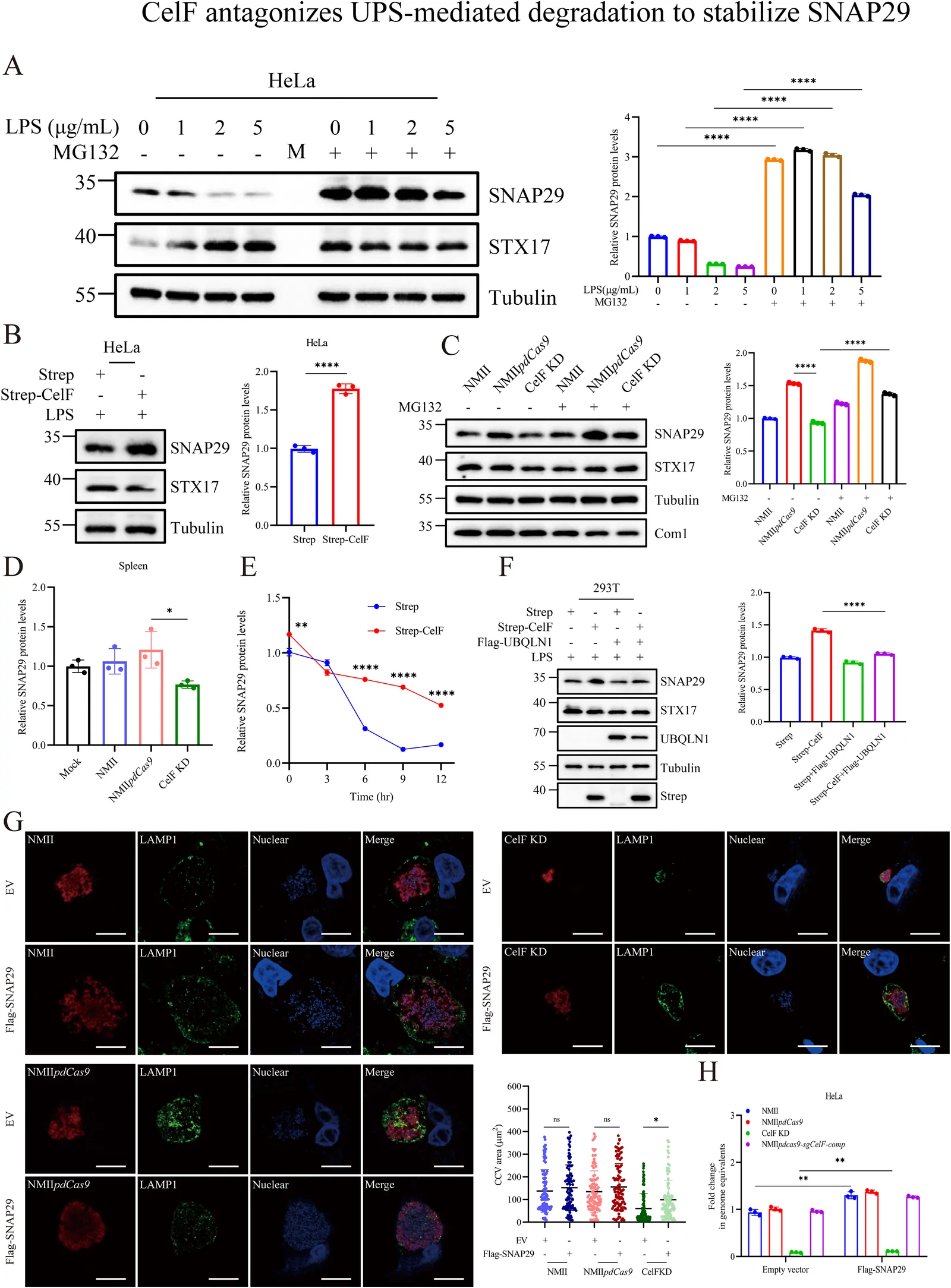
CelF stabilizes SNAP29 by antagonizing UPS-mediated proteolytic degradation. **(A)** HeLa cells were treated with *E. coli* LPS for 24 h and then with the proteasome inhibitor MG132 for 6 h. Cell lysates were collected, and SNAP29 protein levels were analyzed by immunoblotting and quantified. **(B)** HeLa cells transfected with Strep-tagged CelF or an empty vector were treated with *E. coli* LPS. **(C)** HeLa cells infected with the indicated strains (MOI = 20) were treated with MG132. SNAP29 protein levels were analyzed by immunoblotting and quantified. **(D)** SNAP29 protein levels in the spleens of SCID mice (n = 3 mice per group) infected with the indicated strains were analyzed by immunoblotting. **(E)** Protein stability was assessed in *E. coli* LPS-treated HeLa cells transfected with Strep-tagged CelF or an empty vector and treated with cycloheximide (CHX) for the indicated times. **(F)** HEK-293T cells co-transfected with Strep-tagged CelF and Flag-tagged UBQLN1 were treated with *E. coli* LPS. SNAP29 protein levels were analyzed and quantified. **(G)** CCV morphology was assessed in HeLa cells transfected with Flag-tagged SNAP29 and then infected with the indicated strains (MOI = 20) for 3 days. CCV cross-sectional areas were quantified from representative confocal microscopy images showing bacteria (red), LAMP1 (green), and nuclei (blue). Scale bar, 10 µm. n = 100. **(H)** Intracellular replication of *C. burnetii* was assessed in HeLa cells transfected with an empty vector or Flag-tagged SNAP29. Bacterial GEs were quantified at 4 days post-infection. Relative fold changes were calculated relative to those in non-transfected cells. Data are representative of three independent experiments, and bars represent the mean ± SD from three independent experiments. Significance is indicated as follows: ns, *P* ≥ 0.05, *P < 0.05, **P < 0.01, ****P < 0.0001.

Furthermore, heterologous expression of CelF increased the SNAP29 levels (**Figure 5B**; **Supplementary Figure S4B**). To confirm this regulation occurs during infection, we infected HeLa cells with wild-type, control, or CelF KD strains and found that CelF knockdown reduced SNAP29 levels. This decrease was rescued by MG132 treatment, confirming that CelF regulates SNAP29 degradation in a proteasome-dependent manner during bacterial infection (**Figure 5C**). We also examined SNAP29 levels in spleen tissues from infected mice. As shown in **Figure 5D** and **Supplementary Figure S4C**, SNAP29 protein levels are significantly lower in the CelF KD-infected spleens.

To directly assess SNAP29 protein stability, we treated cells with cycloheximide (CHX) to inhibit new protein synthesis. The stability of SNAP29 was significantly increased in CelF-expressing cells (**Figure 5E**; **Supplementary Figure S4D**). This stabilization was dependent on UBQLN1, as the CelF-induced increase in SNAP29 was inhibited by overexpressing UBQLN1 (**Figure 5F**). To determine if CelF acts by preventing SNAP29 ubiquitination, we performed ubiquitination assays, which showed that CelF did not affect the ubiquitination levels of endogenous SNAP29 (**Supplementary Figure S4E**). Consistent with the importance of SNAP29 stabilization, a decrease in SNAP29 protein levels also inhibited C. burnetii proliferation (**Supplementary Figure S4F**). Furthermore, ectopic expression of Flag-tagged SNAP29 in HeLa cells not only promoted the growth of wild-type strains but also partially rescued the intracellular growth and lysosomal metabolic maturation of the CCV defects caused by CelF knockdown (**Figures 5G, H**; **Supplementary Figure S4G**).

### CelF disrupts the UBQLN1-PSMD4 interaction to modulate SNARE proteostasis and lysosomal fusion

The 26S proteasome employs ubiquitin receptors including PSMD4 (also named hRpn10) to capture ubiquitinated targets (Tanaka, 2009; Gadhave et al., 2020). Previous studies have demonstrated that the UBL domain of shuttle factors like UBQLN1 binds PSMD4 (Hamazaki et al., 2015; Harman and Monteiro, 2019). We hypothesized that CelF, by binding the UBL domain of UBQLN1, competitively disrupts its interaction with PSMD4, impairing SNAP29 degradation.

Co-IP assays showed that CelF heterologous expression enhanced UBQLN1-SNAP29 association (**Figures 6A, B**), while reducing UBQLN1-PSMD4 interaction (**Figures 6C, D**). To further validate the role of PSMD4 in SNARE complex assembly, we knocked down PSMD4 using siRNA in HEK-293T cells and observed enhanced binding between SNAP29 and STX17 (**Figure 6E**), consistent with the model that loss of PSMD4 promotes SNARE assembly.

**Figure 6:**
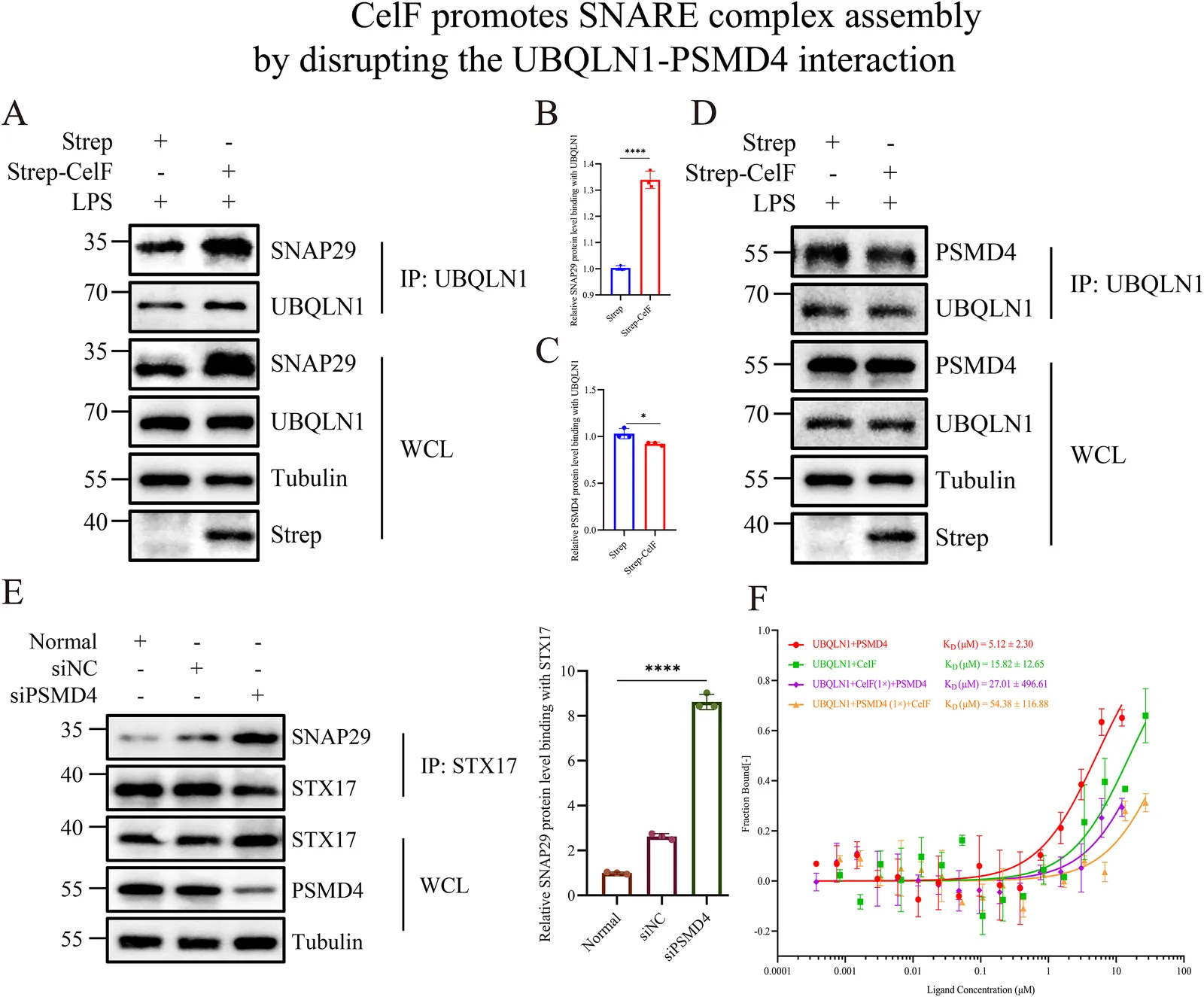
CelF disrupts the UBQLN1-PSMD4 interaction to modulate SNARE proteostasis and lysosomal fusion. **(A–D)** HEK-293T cells transfected with Strep-tagged CelF or an empty vector were treated with *E. coli* LPS. Cell lysates were immunoprecipitated with an anti-UBQLN1 antibody. The interaction of SNAP29 **(B)** or PSMD4 **(C)** with UBQLN1 was quantified. **(E)** HEK-293T cells were transfected with control or PSMD4-targeting siRNA. SNARE complex formation was assessed by Co-IP with an anti-STX17 antibody. Data are representative of three independent experiments, and bars represent the mean ± SD from three independent experiments. Significance is indicated as follows: *P < 0.05, ****P < 0.0001. **(F)** Protein interactions were quantitatively analyzed by microscale thermophoresis (MST). Binding curves show interactions between UBQLN1 and CelF (green), UBQLN1 and PSMD4 (red), and competition between CelF and PSMD4 for UBQLN1 binding (orange and purple). K_D_ values are shown as means ± 95% confidence intervals.

To obtain quantitative evidence for competitive binding, we employed microscale thermophoresis (MST) assays. Binding assays revealed that PSMD4 bound to UBQLN1 with a moderate affinity (KD = 5.12 ± 2.30 µM) (**Figure 6F**; **Supplementary Figure S5A**). Pre-incubating UBQLN1 with CelF before titration with PSMD4 resulted in a significant rightward shift of the binding curve (KD of 27.02 ± 497.61 µM), indicating that CelF influenced the binding of UBQLN1 and PSMD4 (**Figure 6F**; **Supplementary Figure S5B**). Critically, MST analysis also revealed moderate binding of CelF and UBQLN1 (KD = 15.82 ± 12.65 µM) (**Figure 6F**; **Supplementary Figure S5C**). In a competition assay, pre-incubating UBQLN1 with PSMD4 before titration with CelF resulted in the decreased binding affinity of UBQLN1 and CelF due to PSMD4 (KD of 54.38 ± 116.88 µM) (**Figure 6F**; **Supplementary Figure S5D**).

Together, these findings indicate that CelF competitively binds to UBQLN1, shielding it from PSMD4-mediated proteasomal targeting and ultimately facilitating SNARE complex assembly.

## Discussion

In this study, we identify a previously unrecognized mechanism by which an intracellular bacterial effector reprograms host proteostatic metabolism to control lysosomal organelle identity (**Figure 7**). The *C. burnetii* T4SS effector CelF acts as a non-enzymatic molecular decoy that competitively binds the UBL domain of UBQLN1, disrupting its interaction with the proteasome subunit PSMD4. This competitive interference selectively stabilizes the autophagic SNARE protein SNAP29, enhances assembly of the STX17-SNAP29-VAMP8 fusogenic complex, and drives autophagosome-lysosome fusion to support the lysosomal metabolic maturation of the CCV. Our findings expand the current understanding of microbial metabolic subversion strategies and provide novel insights into the physiological regulation of SNARE proteostasis.

**Figure 7:**
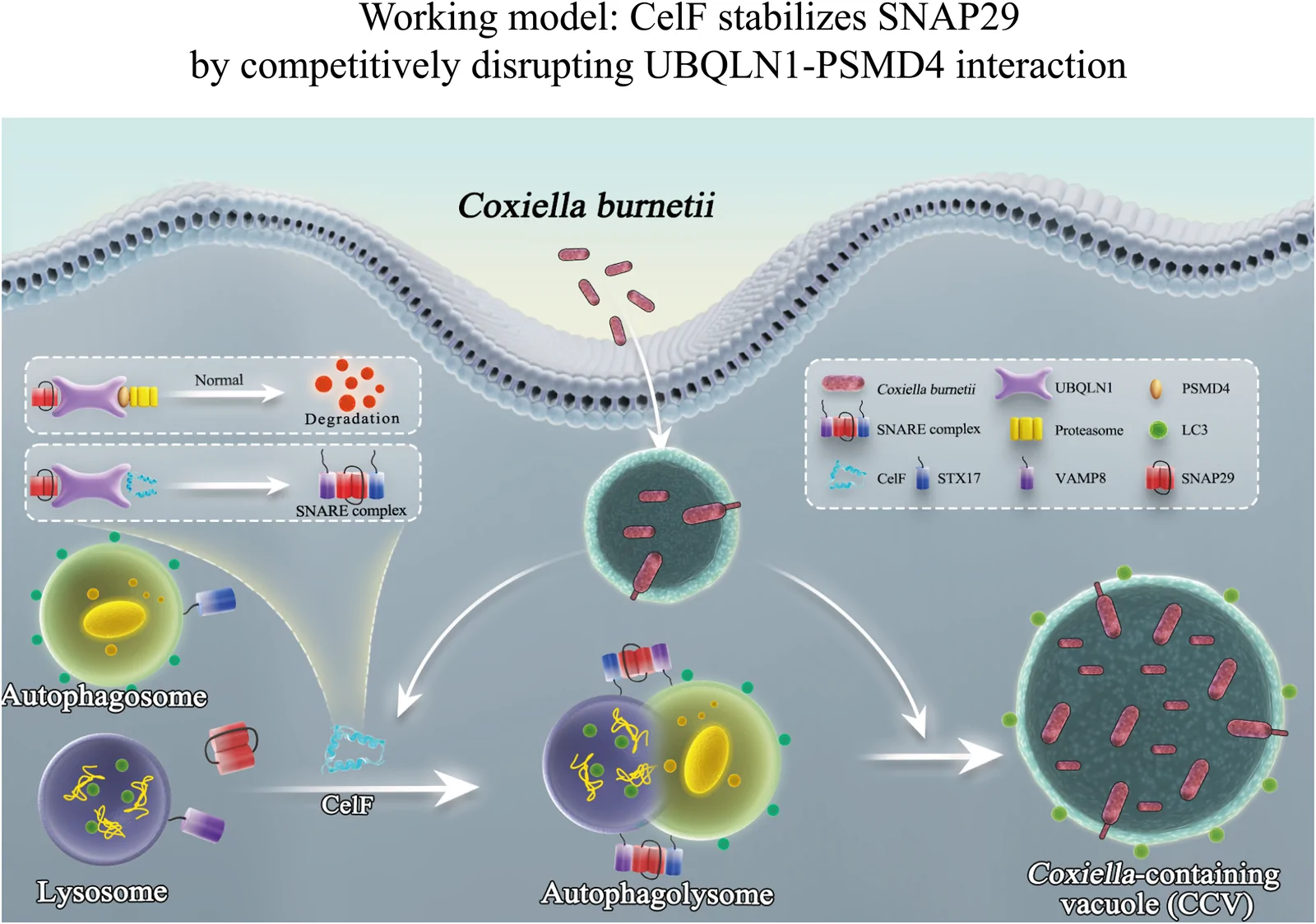
Working model: CelF stabilizes SNAP29 by competitively disrupting UBQLN1-PSMD4 interaction. In uninfected cells (left), UBQLN1 binds ubiquitinated SNAP29 via its UBA domain and delivers it to the proteasome via PSMD4 interaction with its UBL domain, leading to SNAP29 degradation. During *C. burnetii* infection (right), the effector protein CelF is secreted via the T4SS and binds to the UBL domain of UBQLN1, competing with PSMD4. This disrupts SNAP29 delivery to the proteasome, stabilizing SNAP29 and promoting SNARE complex (STX17-SNAP29-VAMP8) assembly. Enhanced SNARE activity drives autophagosome-lysosome fusion, facilitating CCV biogenesis and bacterial replication.

The UPS is a central metabolic system that regulates protein stability and cellular homeostasis, and it is a frequent target of intracellular pathogens. Most known pathogen-encoded UPS modulators exert their functions through enzymatic activities, such as E3 ubiquitin ligases that promote degradation of host defense factors or deubiquitinases that stabilize host proteins beneficial to infection (Mukherjee and Dikic, 2022). For example, *Shigella flexneri* effector IpaH9.8 functions as an E3 ligase to target host GBPs for proteasomal degradation and suppress innate immunity (Li et al., 2017). In stark contrast, CelF does not possess any recognizable enzymatic domain and does not alter the ubiquitination status of its downstream target SNAP29. Instead, it operates purely through competitive protein-protein interaction to block substrate delivery to the proteasome, thereby tuning the metabolic flux of UPS-mediated protein degradation. This non-enzymatic decoy strategy confers several functional advantages from a metabolic regulation perspective. First, it allows selective modulation of a subset of UBQLN1 substrates without globally disrupting proteasomal activity, which would otherwise cause severe metabolic toxicity to the host cell. In support of this, we observed that CelF specifically elevates SNAP29 protein levels without causing global accumulation of polyubiquitinated proteins. Second, competitive inhibition provides a tunable and reversible mode of metabolic regulation, which may be well suited for the gradual maturation process of the CCV. Third, this mechanism represents a remarkable example of convergent evolution at the protein interaction interface, where a bacterial effector mimics the proteasomal binding surface to intercept a host metabolic shuttle factor.

SNARE proteins are the core machinery that mediates intracellular membrane fusion and organelle metabolic communication (Moreau et al., 2013; Fu et al., 2018). While the structural basis of SNARE complex assembly has been extensively studied, the metabolic mechanisms that control SNARE protein stability remain much less understood. Our study identifies SNAP29 as a physiological substrate of UBQLN1-mediated proteasomal degradation, and establishes the UBQLN1-PSMD4 axis as a direct regulatory checkpoint for SNARE proteostasis. This finding has implications beyond microbial pathogenesis: dysregulation of SNAP29 and autophagosome-lysosome fusion has been implicated in metabolic disorders, neurodegenerative diseases, and cancer. The UBQLN1-PSMD4-SNAP29 axis may therefore represent a potential therapeutic target for diseases characterized by impaired autophagic flux and lysosomal metabolic dysfunction.

Notably, the reduction in UBQLN1-PSMD4 binding caused by CelF is quantitatively modest, yet functionally significant. This is consistent with the physiological nature of proteasomal shuttle factor interactions: PSMD4 is a multifunctional proteasomal receptor that engages multiple ligands, including ubiquitin chains, Rpn13, and UBQLN family proteins (Husnjak et al., 2008; Zhang et al., 2009; Kurlawala et al., 2017). Under steady-state conditions, only a fraction of PSMD4 molecules are occupied by UBQLN1. In this equilibrium, even a partial displacement of UBQLN1 from PSMD4 can lead to measurable changes in substrate delivery efficiency and downstream metabolic outputs. The selective stabilization of SNAP29, rather than global proteasome inhibition, further supports the notion that CelF fine-tunes a specific metabolic node rather than broadly suppressing the UPS.

Autophagy is traditionally viewed as a catabolic metabolic pathway that maintains cellular energy homeostasis and quality control (Christian et al., 2013; Galluzzi et al., 2014; Yang et al., 2016). In the context of infection, autophagy serves dual roles: it acts as an innate immune defense mechanism to clear pathogens (Winchell et al., 2016; Siqueira et al., 2018; Wu and Li, 2019), but it can also be co-opted by intracellular microbes to support their metabolic demands. *C. burnetii* represents a paradigmatic example of the latter, as it actively recruits autophagic membranes and promotes autophagosome-lysosome fusion to build its replicative niche (Thomas et al., 2020; Kodori et al., 2023; Wang et al., 2023). Our work provides a mechanistic link between pathogen-induced metabolic reprogramming and the core autophagy fusion machinery. By stabilizing SNAP29, CelF boosts the cellular fusogenic capacity and sustains autophagic flux, which in turn supplies the CCV with membranes and metabolic resources.

This strategy is fundamentally different from that employed by other intravacuolar pathogens. For instance, *Legionella pneumophila* blocks phagosome acidification and avoids lysosomal fusion entirely, whereas *C. burnetii* embraces the lysosomal metabolic environment and even accelerates its maturation (Thomas et al., 2020). The discovery of CelF adds to a growing body of evidence that intracellular pathogens have evolved diverse metabolic strategies to shape their intracellular niches. From a metabolic physiology perspective, studying these pathogen-host interactions not only advances our understanding of infectious diseases but also reveals fundamental regulatory mechanisms of cellular metabolism.

Our study has several limitations that warrant further investigation. First, the mouse experiments did not include the CelF-complemented strain. However, the cell-based complementation data (**Figures 1C, D**) support that the observed phenotypes are specifically attributable to CelF loss. Future *in vivo* validation with a complemented strain is warranted. Second, UBQLN1 is known to recognize a broad range of ubiquitinated substrates. It remains to be determined whether CelF-mediated inhibition affects other UBQLN1 substrates beyond SNAP29, and whether such effects contribute to broader metabolic remodeling during infection. Quantitative proteomic profiling of UBQLN1 substrates in the presence and absence of CelF would help address this question. Third, the atomic details of the CelF-UBQLN1 interaction and the competitive displacement of PSMD4 remain to be elucidated. Structural characterization of these complexes would define the molecular basis of this metabolic decoy mechanism and may facilitate the development of targeted modulators of the UBQLN1-PSMD4 axis. Forth, the specific ubiquitination signals that target SNAP29 for UBQLN1 recognition have not been identified. Deciphering the post-translational modification code of SNAP29 will deepen our understanding of SNARE proteostasis regulation.

In conclusion, our study reveals that the *C. burnetii* effector CelF competitively disrupts the UBQLN1-PSMD4 proteasomal interaction to stabilize the autophagic SNARE SNAP29, thereby promoting lysosomal metabolic maturation of the CCV. This work establishes a non-enzymatic paradigm for microbial manipulation of host proteostatic metabolism and identifies the UBQLN1-PSMD4-SNAP29 axis as a novel physiological regulatory node for SNARE homeostasis and autolysosomal function. These findings advance our understanding of host-pathogen metabolic interactions and provide fundamental insights into the proteostatic control of membrane fusion machinery.

## Materials and methods

### Ethics statement

All procedures for animal experiments were approved by the Institute of Animal Care and Use Committee (IACUC) of the Academy of Military Medical Sciences (approval no. IACUC-DWZX-2024-P006), and all experiments were performed following this committee’s regulations and guidelines.

### Cell lines, bacterial strains and mice

HeLa and HEK-293T cells purchased from ATCC were cultured in DMEM medium containing 10% fetal bovine serum (FBS). THP-1 cells purchased from ATCC were cultured in RPMI 1640 medium with 10% FBS. All cell lines were maintained at 37˚C with 5% CO2. The C. burnetii Nine-Mile phase II strain (NMII) maintained in our laboratory was cultivated in acidified citrate cysteine medium (ACCM-2) with 5% CO2 and 2.5% O2 at 37 ˚C as previously described (Omsland et al., 2011). NMIIpTEM1, NMIIpTEM1-Flag CelF, dotA::Tn, dotA::Tn pTEM1, dotA::Tn pTEM1-Flag CelF, NMIIpdCas9 and NMIIpdCas9-sgCelF strains were generated by our lab and cultured in ACCM-2 medium supplemented with the indicated antibiotics. Severe combined immune deficiency (SCID) mice (aged 6 to 8 weeks) were purchased from the Vital River Laboratory (Beijing, China).

### Generation of *C. burnetii* mutants

The strains described above were generated by electroporation. Briefly, approximately 10 μg of plasmid DNA was electroporated into the *C. burnetii* NMII strain or the *dotA*::Tn strain under the following conditions: 1.8 kV, 25 μF, and 500 Ω, as previously described (Newton et al., 2014). After three passages in ACCM-2 medium containing kanamycin (400 μg/mL) or chloramphenicol (3 μg/mL), positive *C. burnetii* transformants were screened using ACCM-2 agar plates supplemented with the indicated antibiotics. Single colonies of *C. burnetii* mutant strains were selected and cultured for further analysis. The expression of CelF in the NMII*pTEM1*-CelF and *dotA*::Tn *pTEM1*-CelF strains was verified by Western blotting using an anti-FLAG antibody. For the NMII*pdCas9* and NMII*pdCas9*-*sgCelF* (designated CelF KD) strains, the expression levels of *celF* and *dCas9* were determined by reverse transcription quantitative PCR (RT-qPCR) using primers specific for the deleted or introduced genes, with primers targeting *rpoB* as a control. Three independent CelF KD strains were generated using three different sgRNAs (sgRNA1, sgRNA2, and sgRNA3). To generate a complementation strain for the CelF knockdown mutant, *celF* was inserted into the indicated vector to produce NMII*pdCas9*-*sgCelF*-*comp*. These strains were cultured in ACCM-2 medium supplemented with chloramphenicol for 7 days and then used to infect cells at an appropriate multiplicity of infection (MOI).

### *C. burnetii* growth *in vitro* and cell lines

C. burnetii wild-type (WT) or mutant strains were cultivated in ACCM-2 medium with or without the corresponding antibiotics. To assess the growth characteristics in ACCM-2 medium, C. burnetii strains were inoculated into 20 mL of fresh ACCM-2 at a concentration of 1×106 genome equivalents (GEs)/mL. At different time points (1, 2, 3, 4, 5, 6, and 7 days) post-inoculation, 1 mL of each sample was taken for DNA extraction. The C. burnetii GE was quantitated by qPCR targeting the com1 gene as previously described (Fu et al., 2022). To plot the growth curves of C. burnetii in human macrophages, THP-1 cells were seeded in 12-well plates at a density of 1×106/mL and cultured in RPMI 1640 medium containing 1% FBS with 200 nM phorbol myristate acetate (PMA). THP-1 cells were then infected with C. burnetii wild-type (WT) or mutant strains at a multiplicity of infection (MOI) of 5. Four hours post-infection, cells were washed twice with phosphate-buffered saline (PBS) and continually incubated in fresh medium supplemented with 1% FBS. Then cells were collected daily for 7 days. Genomic DNA from C. burnetii-infected cells was extracted and subjected to qPCR to quantify GE by a probe specific to com1.

### TEM translocation assays

TEM translocation assays were performed as described previously with some modifications. Briefly, THP-1 cells were seeded in a 96-well plate at 5×10^4^/mL and cultured in RPMI 1640 medium containing 1% FBS and 200 nM PMA. THP-1 cells were then infected with NMII or *dotA*::Tn strains carrying each of the β-lactamase fusions at an MOI of 20. *C. burnetii* was transformed with the empty vector as a negative control and with CvpE as a positive control (Zhao et al., 2024). Four hours after infection, cells were washed with PBS to remove uninternalized bacteria. Infected cells were loaded with the fluorescent substrate CCF4/AM according to the instructions for the LiveBLAzer-FRET B/G Loading Kit (Invitrogen, K1095) at 24 hours post-infection and incubated at room temperature for 1–3 hours. The ratio of fluorescence signal at 460 nm to 535 nm (blue/green) was calculated relative to uninfected cells, which was quantified using a fluorescence microscope (Olympus).

### Cell transfection

For the ectopic expression of plasmids in mammalian cells, cells were seeded at a density of 1 × 10^5^/mL. Cells were transfected with Lipofectamine™ 8000 transfection reagent (Beyotime) according to the manufacturer’s instructions. At the indicated time points post-transfection, the cells were fixed and processed for immunofluorescence or harvested for Western blotting.

### Antibodies

The antibodies used in this study were mouse anti-Strep-II tag (MBL, M211-3), mouse anti-Flag tag (Sigma, F1084), mouse anti-α-Tubulin (MBL, PM054), mouse anti-GAPDH (Proteintech, 14064-1-AP), mouse anti-His (Proteintech, 66005-1-Ig), rabbit anti-UBQLN1 (CST, 14526S), mouse anti- STSQM1/p62 (CST, 8588S), Rabbit anti-LC3B (CST, 3868S), Rabbit anti-SNAP29 (Proteintech, 12704-1-AP), Rabbit anti-STX17 (Proteintech, 17815-1-AP), Rabbit anti-Ubiquitin (Proteintech, 80992-1-RR), Mouse anti-PSMD4 (Proteintech, 66179-1-Ig). Mouse anti-*C. burnetii*, mouse anti-CelF, and mouse anti-com1 polyclonal antibodies were generated in our laboratory.

### Small interfering RNA transfection

Expression of targeted genes was knocked down by transient transfection of small interfering RNA (siRNA). The targeted sequences purchased from GenePharma were as follows: PSMD4, 5’-AAGGAGGAAGACAAGAAGUGA-3’, UBQLN1, 5’-GGCGCAUGUACACAGAUAU-3’, SNAP29, 5’-GAAGCUAUAAGUACAAGUA-3’. Cells were harvested 48 h after transfection of siRNAs.

### Co-immunoprecipitation

CelF was cloned into an mCherry-Strep-tagged plasmid, and UBQLN1 and its truncations were cloned into FLAG-tagged plasmids. To investigate the interaction between CelF and UBQLN1, HEK-293T cells were co-transfected with plasmids overexpressing CelF, UBQLN1, or an empty vector as a control using Lipofectamine 8000. Twenty-four hours post-transfection, cells were lysed using lysis buffer with protease and phosphatase inhibitor cocktails on ice for 30 min. After centrifugation at 10,000 ×*g* for 5 min to remove the debris, cell lysates were incubated with anti-FLAG antibody at 4 ˚C for 6 hours. Then, 50 μL of a 50% slurry consisting of Protein A/G plus-agarose (Cytiva) was added, mixed and incubated for another 6 hours or overnight. After 5 washes with lysis buffer at 500 g and 4 °C, the samples were boiled with 2×SDS loading buffer for 10 min. Samples were analyzed by Western blotting using anti-FLAG and anti-Strep-II antibodies, followed by an appropriate secondary antibody.

### Pull-down assay

Purified 1 μg His-tagged CelF protein and purified 1 μg His-tagged UBQLN1 protein (generated by CUSABIO, Wuhan, China) were mixed in reaction buffer (50 mM Tris-HCl, 100 mM glycine, and 150 mM NaCl, pH7.5) for 8 h at 4 °C. The mixture was then incubated with 50 μL slurry of Protein A/G plus-agarose conjugating with 1 μg anti-UBQLN1 antibody overnight at 4 °C. Agarose was washed five times with lysis buffer and then boiled in loading buffer for 10 min. Samples were analyzed by western blotting using 1 μg anti-His tag or anti-UBQLN1 antibodies.

### Western blotting

Protein samples were fractionated by electrophoresis on SDS-PAGE gels, and resolved proteins were transferred to polyvinylidene difluoride (PVDF) membranes. After blocking with 5% skim milk, the membranes were incubated with the indicated primary antibodies, followed by appropriate horseradish peroxidase (HRP)-conjugated secondary antibodies. Blots were developed with an enhanced chemiluminescence (ECL) kit.

### Cell imaging

To assess the CCV size of C. burnetii WT or mutant strains during C. burnetii infection, THP-1 cells were seeded onto coverslips in a 24-well plate in the presence of 200 nM PMA. After differentiation, THP-1 cells were infected with NMII, NMIIpdCas9, CelF KD and NMIIpdCas9-sgCelF-comp strains at an MOI of 20. Four days after infection, the cell culture medium was discarded, and the cells were washed 3 times with PBS. Then, the cells were fixed with 4% paraformaldehyde (Beyotime, P0099) for 10 min, permeabilized with 0.2% Triton X-100 (Beyotime, P0096) for 15 min, and then blocked with 1% bovine serum albumin (BSA). Then, cells were probed with an anti-LAMP1 antibody (CST, 9091S) and anti-C. burnetii serum, followed by goat anti-rabbit IgG Alexa Fluor 488 antibody (Abcam, ab150077) or goat anti-mouse IgG Alexa Fluor 594 antibody (Abcam, ab150116). After three washes with PBS, the cell nuclei were stained with DAPI. The coverslips were removed and mounted on slides with Prolong Gold antifade mounting solution (Thermo, P36935). To detect the colocalization of exogenous CelF and UBQLN1 truncation mutants, HeLa cells were seeded in 24-well plates, cultured overnight, and transfected with the indicated eukaryotic recombinant plasmids. After fixation and permeabilization, cells were blocked and probed with an anti-FLAG antibody, followed by treatment with a goat anti-mouse IgG Alexa Fluor 488 antibody. Finally, the cells were incubated with DAPI to stain the nuclei. Coverslips were mounted on slides with Prolong Gold antifade mounting solution. The samples were observed using a spinning disk confocal microscope (Olympus, FV3000). The size of CCVs or colocalization was quantified for fluorescent signals in ImageJ software.

### Detection of assembly of SNARE complex

Cells were cultured in a 6-well plate at a density of 2×10^5^ cells/mL and subjected to plasmid transfection. At the appointed time points, the cell lysates were collected and incubated with anti-STX17 antibody at 4 ˚C for 6 hours. Then, 50 μL of a 50% slurry consisting of Protein A/G plus-agarose was added, mixed and incubated for another 6 hours or overnight. After 5 washes with lysis buffer at 500 ×*g* and 4 °C, the samples were boiled with 2×SDS loading buffer for 10 min. Samples were analyzed by Western blotting using anti-SNAP29 and anti-STX17 antibodies, followed by an appropriate secondary antibody.

### MST assay

All MST measurements were performed on a Monolith NT.115 system (NanoTemper Technologies). Full-length recombinant UBQLN1 (CUSABIO, Wuhan, China) was fluorescently labeled using the Monolith Protein Labeling Kit RED-NHS 2nd Generation (MO-L011, NanoTemper) according to the manufacturer’s protocol. The labeled protein was diluted in assay buffer (PBST containing 0.05% Tween 20) to a working concentration of 20 nM. His-tagged CelF (CUSABIO) and PSMD4 (Solarbio, Beijing) were used as unlabeled titrants and dissolved in the corresponding ligand buffers (PBS with 6% Trehalose, pH 7.4 for CelF; 20 mM Tris-HCl, 150 mM NaCl, 1 mM DTT, pH 7.5 for PSMD4).

For direct binding affinity measurements, two-fold dilution series of CelF or PSMD4 were prepared in corresponding buffers. Subsequently, 10 μL of each dilution was mixed with 10 μL of the 20 nM labeled UBQLN1 solution, yielding final concentrations of 27.2 µM-0.83 nM for CelF and 12.2 µM-0.372 nM for PSMD4 in a 20 µL assay mixture. A buffer control without unlabeled ligand was included in parallel. The mixtures were incubated at 25 °C for 10 min in the dark and loaded into hydrophilic standard glass capillaries (NanoTemper Technologies) for MST measurement.

For competition MST experiments, labeled UBQLN1 was first pre-incubated with a fixed concentration of the competing protein in assay buffer at 25 °C for 10 min in the dark. The competing protein was used at a final concentration of 5.12 µM for PSMD4 or 15.82 µM for CelF. Then, 10 µL of the pre-incubated mixture was mixed with 10 µL of a two-fold dilution series of the titrant (CelF or PSMD4), yielding final titrant concentrations of 27.2 µM-0.83 nM for CelF and 12.2 µM-0.372 nM for PSMD4 in the 20 µL assay mixture. The final concentrations of labeled UBQLN1 and the competing protein were 20 nM and 5.12 µM (PSMD4) or 15.82 µM (CelF), respectively. The samples were loaded into hydrophilic standard glass capillaries (MO-k022) and measured on the Monolith NT.115Pico system.

Data were analyzed using MO.Affinity Analysis software (NanoTemper Technologies) to derive the equilibrium dissociation constant (K_D_). Signal-to-noise ratios were >5 in all measurements.

### Quantitative analysis of *C. burnetii*

Total DNA was extracted from ACCM-2 cultured C. burnetii or C.burnetii-infected cells using a DNeasy Tissue kit following the manufacturer’s instructions. Taqman Universal Master Mix was used to perform real-time PCR reactions in duplicates on Applied Biosystems QuantStudio 3. Diluted pQGK-com1 plasmid was used as the standard sample. The primers and probes used for quantification were as follows: com1-F: 5’-aaaacctccgcgttgtcttca-3’; com1-R: 5’-gctaatgatactttggcagcgtattg-3’; com1-probe: 5’-FAM-AGAACTGCCCATTTTTGGCGGCCA-BHQ1-3’.

### Reverse transcription quantitative PCR (RT-qPCR)

NMII, NMIIpdCas9, CelF KD and NMIIpdCas9-sgCelF-comp strains were used with the PureLink™ RNA Mini Kit following the manufacturer’s instructions. RT-qPCR was performed using the One Step TB Green PrimeScript™ PLUS RT-PCR Kit and Applied Biosystems QuantStudio 3. The specific primers used for CelF (sense primer: 5’- CAAGTCCTACATCAAGGGAGCC-3’ and antisense primer: 5’- TATCCGCTTTTGCCCCTATCG-3’) and dcas9 (sense primer: 5’- tgcgtatctaaatgccgtcgttgg-3’ and antisense primer: 5’-ttgcggttgctttgcctatttcttg-3’) were synthesized by Sangon Company (Suzhou, China). The rpoB gene (sense primer: 5’-cgtcatttccaccatcgtccctatc-3’ and antisense primer: 5’- gcccaacccagatgagtttccag-3’) was used as the reference gene.

### Animal experiments

To examine the effect of CelF on C. burnetii pathogenicity, 6-week-old female SCID mice (n = 6) were infected with NMII, NMIIpdCas9 or CelF KD strains at a dose of 2×106 GE per mouse via intraperitoneal injection, respectively. Mice were sacrificed at two weeks post-infection. The spleens of each mouse were removed and weighed to determine the degree of splenomegaly (spleen weight/body weight). Subsequently, each spleen was bisected: one half was snap-frozen in liquid nitrogen for DNA extraction, and the other was homogenized in lysis buffer supplemented with protease and phosphatase inhibitors for protein extraction. The GEs of C. burnetii in spleens were determined using qPCR. For the protein analysis, the concentration was determined using a BCA protein assay kit (Thermo Fisher) and detected by Western blotting. All procedures for animal experiments were approved by the Institute of Animal Care and Use Committee (IACUC) of the Academy of Military Medical Sciences (approval no. IACUC-DWZX-2024-P006), and all experiments were performed in accordance with the regulation and guidelines of this committee. 

### Statistical analysis

Differences in *C. burnetii* GE, *C. burnetii* mRNA levels, CCV size, spleen weight-to-body weight ratio, band density and fluorescence intensity were analyzed for statistical significance using Student’s *t-*test, one-way or two-way analysis of variance (ANOVA). Data from all experimental groups were obtained from three independent experiments and are presented as means ± standard deviation (SD), except for *Pearson* correlation coefficients, which are presented as means ± standard error of the mean (SEM). Data were analyzed using GraphPad Prism 8.0 software. For all analysis, *P* < 0.05 was considered statistically significant.

## Data Availability

The original contributions presented in the study are included in the article/**Supplementary Material**. Further inquiries can be directed to the corresponding authors.
